# Upregulation of miR-181c inhibits chemoresistance by targeting *ST8SIA4* in chronic myelocytic leukemia

**DOI:** 10.18632/oncotarget.11054

**Published:** 2016-08-04

**Authors:** Lifen Zhao, Yan Li, Xiaobo Song, Huimin Zhou, Nana Li, Yuan Miao, Li Jia

**Affiliations:** ^1^ College of Laboratory Medicine, Dalian Medical University, Dalian, Liaoning Province, China; ^2^ Department of Clinical Laboratory, the First Affiliated Hospital of Dalian Medical University, Dalian, Liaoning Province, China; ^3^ Department of Medical Biology, Faculty of Health Sciences, University of Tromsø, Tromsø, Norway; ^4^ Department of Microbiology, Dalian Medical University, Dalian, Liaoning Province, China

**Keywords:** miR-181c, ST8SIA4, chronic myelocytic leukemia, PI3K/AKT, chemoresistance

## Abstract

Chemotherapy resistance frequently drives tumor progression. Increased expression of ST8SIA4 has been reported in diverse carcinomas and highly correlates with leukemia multidrug resistance (MDR). MicroRNAs (miRNA) are widely recognized as key players in cancer progression and drug resistance. Here, to explore whether miRNA modulates the sensitivity of chronic myelocytic leukemia (CML) to chemotherapeutic agents and regulates ST8SIA4 expression, we analyzed the complete miRNA expression profile and found a subset of miRNAs specifically dysregulated in adriamycin-resistant CML cell line K562/ADR and its parent cell line K562. Compared with three pairs of CML cell lines and 38 clinical samples of peripheral blood mononuclear cells (PBMC) of CML patients, miR-181c expression was down-regulated in drug-resistant cell lines and CML/MDR samples. Altered expression levels of miR-181c influenced the MDR phenotypes of K562 and K562/ADR. Reporter-gene assay showed that miR-181c directly targeted and inhibited the *ST8SIA4* expression, as well as miR-181c was inversely correlated with the levels of *ST8SIA4* expression in CML cell lines and samples. Moreover, ST8SIA4 could reverse the effect of miR-181c on drug resistance in K562 and K562/ADR cells *in vitro*. Upregulation of miR-181c sensitized K562/ADR cells to adriamycin *in vivo* through directly suppressing ST8SIA4 expression. Further investigation showed that miR-181c mediated the activity of phosphoinositide-3 kinase (PI3K)/AKT signal pathway, and inhibition of PI3K/Akt in K562 cells counteracted miR-181c-mediated MDR phenotype. These data revealed an important role for miR-181c in the regulation of chemoresistance in CML, and suggested the potential application of miR-181c in drug resistance treatment.

## INTRODUCTION

Chronic myeloid leukemia (CML) is a myeloproliferative disorder characterized by BCR-ABL fusion gene [1]. Over the past two decades, major advances have been achieved in the treatment of cancers thanks to the highly potent antineoplastic drugs. However, chemoresistance is still a significant obstacle for successful chemotherapy of CML patients.

MicroRNAs (miRNAs) are a class of regulatory non-coding RNAs of 19-25 nucleotides which act by targeting specific messenger RNAs (mRNAs) for degradation or inhibition of translation through base pairing to partially or fully complementary sites [2]. It has been demonstrated that miRNA-mediated gene regulation was involved in multiple biological processes, such as cell proliferation, migration and invasion, differentiation, survival, and tumorigenesis [3]. It has been reported that miRNAs play important roles in chemotherapeutic resistance [4], highlighting miRNAs as potent therapeutic targets or chemoresistant modulators in cancer treatment. Ectopic expression of miR-370 sensitized K562 cells to homoharringtonine and partially targeted FoxM1 by inducing apoptosis [5]. Additionally, inhibition of miR-21 by antagomiR-21 markedly increased apoptosis induced by imatinib in CML [6], and forced expression of miR-217 sensitizes dasatinib-resistant K562 cells to dasatinib [7]. More recently, miR-17 and miR-20a have been demonstrated to be involved in resistance of the leukemia cells to chemotherapeutic drug VP-16 mediated by BIM-S [8].

Sialic acids are negatively charged nine-carbon carboxylated monosaccharides on glycosylated proteins and lipids formed due to post translational modification [9]. Most cell surface glycans are highly sialylated and often involved in cell-cell and/or cell-extracellular matrix interaction [10]. High level of ST3Gal III sensitizes ovarian cancer cells to Taxo [11]. Endogenous expression of ST6GalNAc I in CML cell line K562 was associated with the expression of the STn O-glycan related to a lack of response to chemotherapy [12]. In many tumors, continued activation of phosphatidylinositol-3-OH kinase (PI3K)-Akt pathway has been implicated as a mechanism of resistance to cytotoxic chemotherapy agents [13, 14]. ST8SIA4 has been reported to enhance the chemoresistance in leukemia by phosphorylating and activating of Akt at Ser473 and Thr308 specifically and increasing the activity of PI3K/AKT signaling pathway [15, 16].

By combining microarray analyses and real-time PCR, we found that miR-181c levels remained low in K562/ADR cells but became markedly higher in K562 cells. Other study has reported that upregulation of miR-181c contributes to chemoresistance in pancreatic cancer. Based on the above, we selected miR-181c for analysis. By miRNA target prediction algorithms, and experimental validation approaches, the objective of this study was to reveal the molecular mechanisms of miR-181c in adriamycin-resistant CML cancer.

## RESULTS

### MiRNA-181c is downregulated in chemoresistant CML cell lines and CML/MDR patients

**Figure 1:**
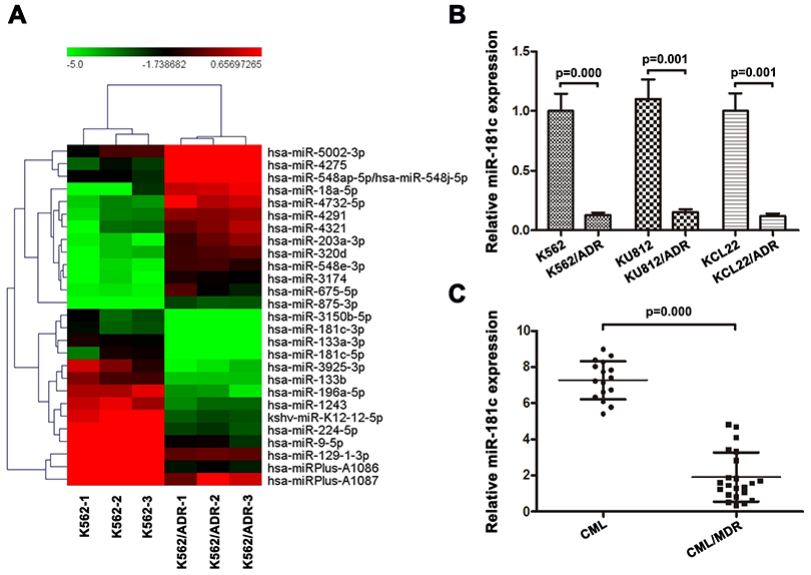
Expression of miR-181c is associated with chemoresistance in chronic myelocytic leukemia **A.** Comparison of miRNA expression in K562/ADR and its parental cells by using the Exiqon Human miRNA microarray. Expression levels of miR-181c in K562 cells were higher than those in K562/ADR cells. **B.** A validation experiment was carried out using qRT-PCR. The relative levels were normalized to U6 snRNA. Expressions of miR-181c in parental K562, KCL22 and KU812 cells were higher compared to their ADR-resistant cells (*p<0.05). **C.** Moreover, chemoresistant cancer samples have significantly lower levels of miR-181c (*p<0.05).

A screen to identify miRNAs involved in adriamycin resistance was performed in K562 and K562/ADR cell lines, which were made adriamycin-resistant by continuous exposure to adriamycin *in vitro*. We identified 41 miRNAs to be dysregulated at least 5-fold in resistant compared to adriamycin sensitive parental cells (Table 1). Supervised hierarchical clustering using the 41 differentially expressed miRNAs clustered cell lines according to miRNA expression rather than doxorubicin response (Figure 1A). We found that miR-181c levels remained low in K562/ADR cells but became markedly higher in K562 cells. We further confirmed miR-181c expression using real-time PCR. As shown in Figure 1B, the expressions of miR-181c were decreased in K562/ADR, KU812/ADR, KCL22/ADR cells. To identify the expression level of miR-181c in CML patients, the PBMC isolated from CML patients was also analyzed. The PBMC were first divided into two groups, CML without MDR and CML/MDR. The frequency of P-gp positivity was 57.9% (22 of 38) in the CML patients. As shown in Figure 1C, miR-181c expression was increased in CML patients compared with that in CML/MDR patients. We hypothesized that miR-181c might functionally regulate therapy response. We therefore focused on its role in chemoresistance regulation.

**Table 1:** A Upregulation of miRNAs in K562/ADR cells by miRNA expression arrays

miRNA Name	Fold change	P value
hsa-miR-493-5p	120.2045211	0.006613234
hsa-miR-4536-3p	119.6133579	0.000324181
hsa-miR-597-5p	86.24456259	0.000283711
hsa-miR-4701-5p	69.54030647	0.00014616
hsa-miR-487b-3p	65.46857073	5.28443E-05
hsa-miR-3142	41.80426403	6.53848E-07
hsa-miR-138-1-3p	40.45461722	3.26891E-05
ebv-miR-BART3-3p	33.98891047	0.000689351
hsa-miR-508-5p	30.4905756	2.87849E-05
hsa-miR-4797-5p	29.61896953	0.002158594
hsa-miR-431-3p	26.73736251	0.010255919
hsa-miR-4699-5p	25.92966162	0.000956889
hsa-miR-570-3p	18.69018818	0.023155274
kshv-miR-K12-10a-5p	17.19981184	0.00512325
hsa-miR-4732-5p	17.06701472	0.006063631
hsa-miR-4275	16.57943021	0.000223334
hsa-miR-18a-5p	16.17461375	0.000607673
hsa-miR-5002-3p	15.10828496	8.31579E-05
hsa-miR-203a-3p	14.87554644	0.004490546
hsa-miR-548e-3p	13.56752185	0.000137616
hsa-miR-875-3p	11.12031471	0.000225273
hsa-miR-320d	10.80688128	0.000182227
hsa-miR-3174	10.77441988	0.000116304
hsa-miR-4291	9.685551326	5.06551E-05
hsa-miR-675-5p	9.524532198	0.022556638
hsa-miR-4321	8.735543826	0.008010134
hsa-miR-548ap-5p/hsa-miR-548j-5p	6.676467834	1.18179E-06

B Downregulation of miRNAs in K562/ADR cells by miRNA expression arrays		
miRNA Name	Fold change	P value
hsa-miR-129-1-3p	0.104514611	0.003460001
hsa-miR-9-5p	0.101656616	4.95002E-06
hsa-miR-1243	0.099436119	0.003384493
hsa-miR-3150b-5p	0.098993831	0.037179275
hsa-miR-133b	0.096682471	0.002976551
hsa-miR-181c-3p	0.086957031	0.020925852
kshv-miR-K12-12-5p	0.085727147	0.005103428
hsa-miR-133a-3p	0.078645918	0.000161493
hsa-miR-181c-5p	0.074656377	0.040297289
hsa-miR-196a-5p	0.061691897	0.002390692
hsa-miR-3925-3p	0.058727961	0.033063265
hsa-miR-224-5p	0.044212601	0.005407617
hsa-miRPlus-A1087	0.037110028	0.000674487
hsa-miRPlus-A1086	0.014841003	0.00048885

### MiR-181c signature impacts chemoresistance of K562 and K562/ADR cells *in vitro*

**Figure 2:**
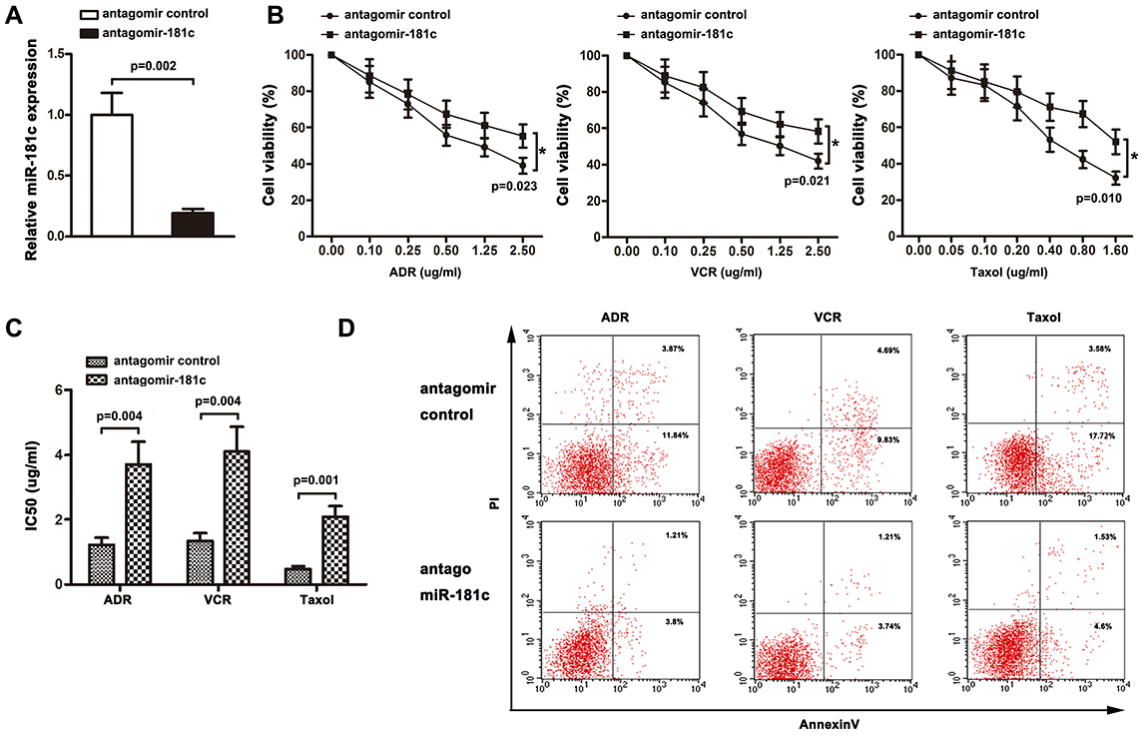
Knockdown of miR-181c promotes drug resistance *in vitro* **A.** qRT-PCR was performed to measure miR-181c levels in K562 cells transfected with the antagomir control or antagomiR-181c. Decreased miR-181c levels were observed in the antagomiR-181-transfected cells compared to the control (*p<0.05). **B.** K562 cells were treated with ADR, VCR and Taxol for 48 h. Cell viability was determined using a CCK8 assay. More cells survived in antagomiR-181c-transfected group (*p<0.05). **C.** Knockdown of miR-181c increased IC50 values of K562 cells to ADR, VCR, and Taxol (*p<0.05). **D.** K562 cells were treated with ADR, VCR and Taxol, followed by analysis of apoptosis. There were fewer cells undergoing apoptosis in the antagomiR-181-transfected group (*p<0.05).

**Figure 3:**
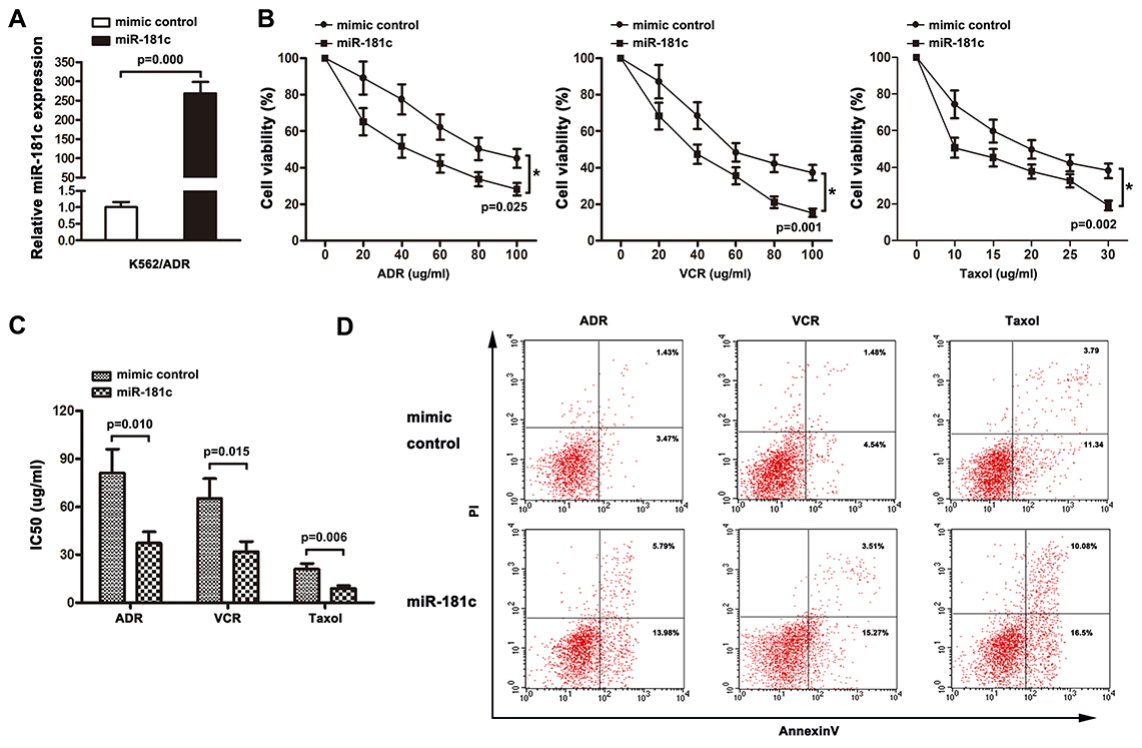
Upregulation of miR-181c decreases cell chemoresistance *in vitro* **A.** qRT-PCR was performed to measure miR-181c levels in K562/ADR cells transfected with the mimic control or miR-181c. Increased miR-181c levels were observed in the miR-181-transfected cells compared to the control (*p<0.05). **B.** K562/ADR cells were treated with ADR, VCR and Taxol for 48 h. Cell viability was determined using a CCK8 assay. Lower cells survived in miR-181c-transfected group (*p<0.05). **C.** Ectopic expression of miR-181c sensitized K562/ADR cells to ADR, VCR and Taxol (*p<0.05). **D.** K562/ADR cells were treated with ADR, VCR and Taxol, followed by analysis of apoptosis. There were more cells undergoing apoptosis in the miR-181c-transfected group (*p<0.05).

The role of miR-181c in chemoresistance was characterized by endogenously silencing miR-181c in K562 cells. As shown in Figure 2B, transient transfection of antagomiR-181c in K562 cell resulted in an increase in cell viability to ADR, VCR and Taxol. Moreover, increased IC50 values for chemotherapeutic agents were observed, after downregulation of miR-181c (Figure 2C). In addition, flow cytometry analysis showed that inhibition of miR-181c led to a decrease in the apoptosis rate (Figure 2D). The role of miR-181c in chemoresistance was further examined by upregulation of miR-181c. K562/ADR cells were transiently transfected with miR-181c or mimic control. Quantitative RT-PCR confirmed that the transient transfection of miR-181c effectively enhanced the expression of miR-181c (Figure 3A). Conversely, transient transfection of miR-181c resulted in a significant decrease in cell viability of K562/ADR cells to ADR, VCR and Taxol (Figure 2B). Furthermore, overexpression of miR-181c sensitized cells to chemotherapy, as indicated by a decrease in the IC50 (Figure 3C). Moreover, flow cytometry analysis confirmed that miR-181c significantly increased the apoptosis of these cancer cells upon drug treatments (Figure 3D). Collectively, these results indicated that downregulation of miR-181c promotes chronic myelocytic leukemia cell resistance to chemotherapeutic drugs *in vitro*.

### *ST8SIA4* is a target of miR-181c in CML cells

To investigate the molecular mechanisms of how miR-181c decreases chemoresistance resistance, several well-developed miRNA algorithms were employed, such as TargetScan, PicTar, and miRNA.org, to obtain possible mRNA targets of miR-181c. *ABI1, ACSL1, APOO, BRAP, BRD1, PLEK,SETX, RLF, ST8SIA4, DHX57, DOCK7, ENPP1, FOS* et al were among the potential targets. Polysialylation of NCAM is implemented by the two polysialyltransferases (polySTs) ST8SIA2 and ST8SIA4. Recently, the phosphatidylinositol-3-kinase (PI3K) also has been implicated in the signalling events initiated by NCAM [15]. ST8SIA4 seems to be the major polyST of the adult brain [16]. Moreover, constitutive activation of PI3K/Akt is associated with the expression of ST8SIA4 [17, 18]. In addition, our previous study has demonstrated it was involved in the development of multidrug resistance in human leukemia [19, 20]. So, among the search results, *ST8SIA4* captured our attention.

Then, we investigated whether *ST8SIA4* was a target of miR-181c in CML cells. Luciferase reporter constructs were made, containing the putative binding sites of *ST8SIA4*-wt-3′UTR(222-243) regions, *ST8SIA4*-wt1-3′UTR(2117-2140) regions or the mutant 3′-UTR regions (mut:*ST8SIA4*-mut-3′UTR(222-243), mut1:*ST8SIA4*-wt1-3′UTR(2117-2140)) of these transcripts within miR-181c–binding seed regions.

**Figure 4:**
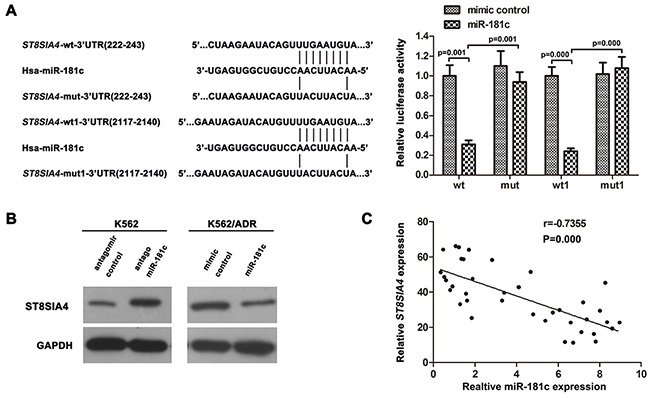
*ST8SIA4* is targeted by miR-181c **A.** Predicted miR-181c target sequence in 3′UTRs of *ST8SIA4* at two different sites. 293T cells were co-transfected with miR-181c or the control and luciferase reporter constructs or the mutants. MiR-181c repressed the activity of *ST8SIA4*-wt-3′UTR and *ST8SIA4*-wt1-3′UTR but had no effect on that of *ST8SIA4*-mut-3′UTR and *ST8SIA4*-mut1-3′UTR (*p<0.05). wt:*ST8SIA4*-wt-3′UTR(222-243), mut:*ST8SIA4*-mut-3′UTR(222-243), wt1:*ST8SIA4*-wt1-3′UTR(2117-2140), mut1:*ST8SIA4*- mut1-3′UTR(2117-2140). **B.** Western blotting of ST8SIA4 expression. GAPDH served as the loading control. **C.** The correlation between miR-181c and *ST8SIA4* expression levels in CML/MDR (N = 22) and CML patients (N = 16)

Forced miR-181c expression decreased luciferase activity, and this suppression was reversed by the mutation of the target sequences in the 3′-UTR of *ST8SIA4* (Figure 4A). Western blotting analysis showed that the inhibition of miR-181c increased ST8SIA4 expression levels in K562 cells. Whereas, ectopic expression of miR-181c by transient transfection decreased the protein expression of ST8SIA4 in K562/ADR cells (Figure 4B). Furthermore, analysis of miR-181c and *ST8SIA4* expression in CML patients by Spearman's correlation analysis showed a significant inverse correlation (r= −0.7355, P =0.000; Figure 4C) Taken together, the results indicated that miR-181c targets *ST8SIA4* to repress its expression by binding to 3′-UTR regions.

### *ST8SIA4* reverses the effect of miR-181c-mediated chemoresistance in K562 and K562/ADR cells

**Figure 5:**
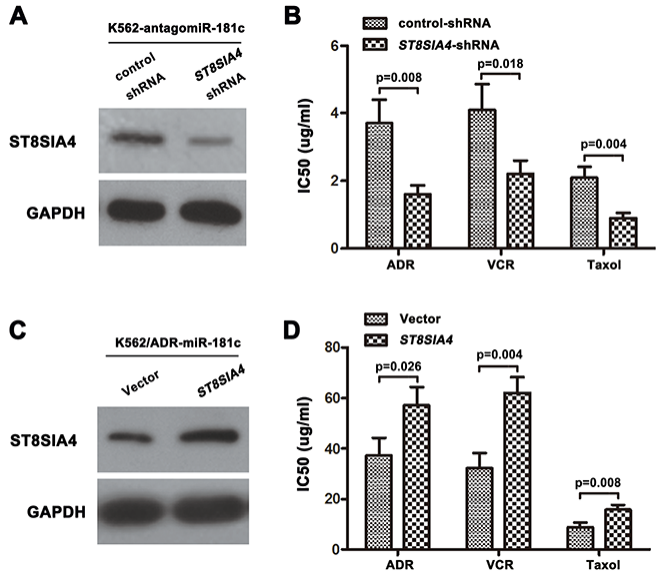
The silence or overexpression of *ST8SIA4* reversed the effect of miR-181c on drug resistance **A, C.** Western blotting analysis of the protein levels of ST8SIA4 in cells transfected with specific *ST8SIA4*-shRNA or *ST8SIA4*, respectively. (B) *ST8SIA4* silencing reversed the drug resistance caused by the downregulation of miR-181c in K562 cells (*p<0.05). **D.** Re-expression of *ST8SIA4* could antagonize miR-181c mediated chemo-sensitivity in K562/ADR cells (*p<0.05).

To assess the possible role of ST8SIA4 in the chemoresistance-mediating capability of miR-181c, we performed functional loss assays by co-transfecting *ST8SIA4*-shRNA plasmid and antagomiR-181c in K562 cells. Western blotting analyses showed a decrease of ST8SIA4 protein in antagomiR-181 transfected clone (K562-antagomiR-181c) (Figure 5A), indicating the effective inhibition of the wild-type protein. While suppression of miR-181c expression promoted chemoresistance, knockdown of *ST8SIA4* reversed antagomiR-181c-mediated sensitivity of the K562 cells to ADR, VCR and Taxol (Figure 5B). Re-introducing the wild-type form of *ST8SIA4* in K562/ADR cells led to a marked increase in the protein expression upon transfection with the miR-181c compared to control (Figure 5C). As shown in Figure 5D, while miR-181c expression induced inhibitory effects on chemoresistance, recombinantly expressed ST8SIA4 reversed miR-181c-mediated sensitivity of K562/ADR cells to drug treatment (Figure 5D). Equally important, knockdown of ST8SIA4 copied the phenotype of high miR-181c levels by sensitizing cells to chemotherapeutic agents [19]. These results suggested that ST8SIA4 is responsible for mediating the effects of miR-181c on chemoresistance in CML cell lines.

### MiR-181c regulates chemoresistance via *ST8SIA4* dependent PI3K/AKT signaling pathway

**Figure 6:**
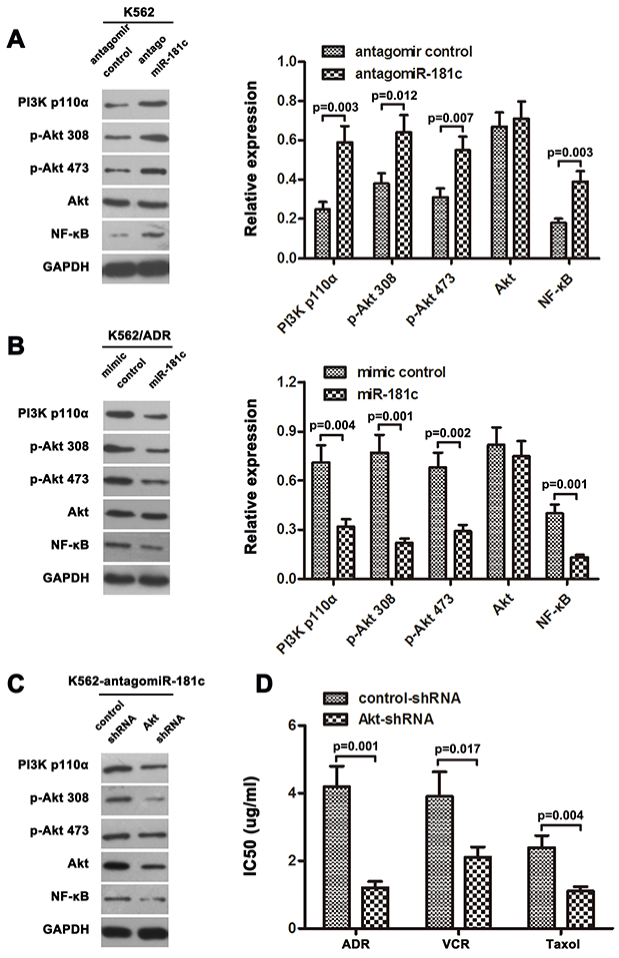
Downregulation of miR-181c activated PI3K/AKT signaling **A, B.** Western blotting analysis of PI3K p110α, phosphorylated Akt (p-Akt 308), phosphorylated Akt (p-Akt 473), total Akt, and NF-κB protein levels in indicated cells. GAPDH was used as a loading control. **C.** Expression of PI3K/Akt signaling molecules were examined by western blot analysis, treated with Akt shRNA in K562/antagomiR-181c cells. **D.** Inactivation of PI3K/Akt signaling using Akt-shRNA significantly increased the chemo-sensitivity of K562 cells transfected with antagomiR-181c, analyzed by CCK-8 assay (*p<0.05).

It has been reported that inhibition of PI3K/AKT signaling has proven to be an efficient way to attenuate the resistance of chemotherapy [36]. Our previous results showed that PI3K/AKT pathway is activated in K562/ADR cells compared with its parental cells K562, and *ST8SIA4* was positive regulators of PI3K/AKT pathway. In present study, given that the expression of ST8SIA4 was down-regulated by miR-181c in CML cells (Figure 4), we further investigated whether dysregulation miR-181c altered the activation of PI3K/AKT pathway. As shown in Figure 6A, a significant increase in PI3K p110α, phospho-Akt 308, phospho-Akt 473 and NF-κB protein was observed when miR-181c was inhibited in K562 cells. By contrast, there was no change in the total amount of Akt protein, demonstrating a truedecrease in phosphorylation status. In addition, after overexpression of miR-181c in K562/ADR cells, we observed a noticeable decrease in protein expression of PI3K110α, p-Akt 308, p-Akt 473 and NF-κB (Figure 6B). Together, the data confirmed that augmented PI3K/AKT pathway expression accompanying miR-181c deficiency was also associated with activation of PI3K/AKT.

To further support the role of PI3K/AKT signaling in regulation chemoresistance by miR-181c, Akt shRNA was utilized in K562 cells. The protein levels of the main signal molecules of PI3K/AKT pathway were analyzed by western blotting. Our results indicated that in K562-antagomiR-181c cells, the protein levels of PI3K/AKT pathway were decreased in Akt shRNA treatment group compared to control group (Figure 6C). In addition, inhibition of PI3K/AKT pathway made the K562-antagomiR-181c cells susceptible to chemotherapy (Figure 6D). These data indicated the involvement PI3K/AKT pathway in suppression of drug resistance by miR-181c.

### MiR-181c decreases chemoresistance of K562/ADR cells *in vivo*

**Figure 7:**
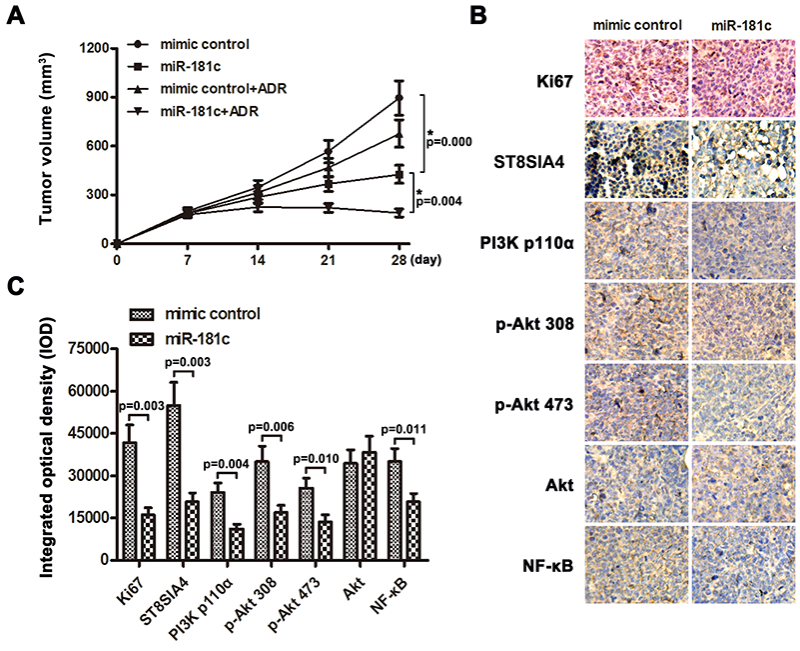
MiR-181c sensitizes K562/ADR cells to chemotherapeutic drugs *in vivo* **A.** Tumor volumes of tumors in the mimic control, mimic control + ADR, miR-181c and miR-181c+ADR groups were measured on indicated days. Data presented are the mean ± SD **B, C.** Expression levels of Ki67, ST8SIA4 and PI3K/Akt signaling molecules in K562/ADR-mimic control group and K562/ADR-miR-181c group were analyzed by immunohistochemistry (*p<0.05).

The promotive effect of miR-181c on CML cell chemoresistance was further examined *in vivo*. Mice were inoculated subcutaneously (1 × 10^7^ K562/ADR cells per mouse) in the right flank. One week later, the mice were randomly divided into four groups (n = 6/group, mimic control group, mimic control+ADR group, miR-181c group, miR-181c+ADR group). Mice were intratumorally injected with mimic control or miR-181c mimic three times per week for three weeks,combining with intraperitoneal injection of adriamycin (7mg/kg) weekly or PBS. The tumor volumes were decreased in the miR-181c group,as compared to the mimic control group (Figure 7A). Significantly, the combined miR-181c and adriamycin treatment markedly restricted the tumor growth to low volumes. These results suggested that miR-181c decreased adriamycin resistance and tumor growth, while injection of miR-181c sensitized K562/ADR cells to adriamycin treatment and inhibited tumor growth. Meanwhile, tumors injected with miR-181c had decreased Ki67, ST8SIA4, PI3K p110α, p-Akt 308, p-Akt 473 and NF-κB expression, analyzed by immunohistochemistry (Figure 7B and 7C). These results further suggest that miR-181c and its downstream PI3K/AKT pathway play important roles in controlling K562/ADR cells adriamycin sensitivity *in vivo*.

## DISCUSSION

MiRNAs can regulate various biological processes in tumorigenesis, metastasis and chemoresistance. In the current study, we reported that specific miRNA expression signatures characterized and contributed to the MDR phenotypic of CML cell lines. Among them, we validated miR-181c, which was downregulated in chemoresistant CML cell lines and CML/MDR patients. Furthermore, miR-181c could exert chemoresistant functions and impede CML drug resistance, partly through targeting *ST8SIA4* and its downstream PI3K/AKT pathway both *in vitro* and *in vivo*. Our data provide a insight into the function of miR-181c in regulating *ST8SIA4* and drug resistance in CML cells.

The miR-181 family contains four miRNAs (miR-181a/b/c/d). Recent studies showed that miR-181 also play a vital role in chemotherapeutic resistance. Restoration of miR-181a expression could sensitize K562/A02 and HL-60/Ara-C cell lines to chemotherapeutic agents [21, 22]. MiR-181b enhanced resistance to the anticancer drug doxorubicin in hepatocellular carcinoma (HCC) [23], and functioned as a tumor suppressor in AML chemoresistance [24]. In the present study, we performed miRNA expression profiling in adriamycin-sensitive cell line K562 and adriamycin-resistant cell line K562/ADR. The expression of miR-181c was decreased in ADR-resistant cell lines (K562/ADR, KU812/ADR, KCL22/ADR) as well as in CML/MDR patients. Moreover, we found that inhibition of miR-181c may enhance resistance to the anticancer drugs of K562 cells. Overexpression of miR-181c sensitized K562/ADR cells to chemotherapy both *in vitro* and *in vivo*. Our findings were supported by investigations in another study. Contrary to our results, miR-181c contributed pancreatic cancer cell resistance to chemotherapeutic drugs by inactivating the Hippo signaling pathway [25]. Down-regulation of miR-181c was found in imatinib-resistant CML patients, compared with imatinib-responders patients [26], which was consistent with our study. These results additionally demonstrate that miR-181c may be a reasonable approach to improving or prolonging drug sensitivity in CML.

Altered mRNA expression levels of sialyltransferases in different cancers are reported as potential targets for therapeuticapproaches [11, 27–29]. Sialyltransferases of the mammalian ST8SIA family catalyze oligo- and polysialylation of surface-localized glycoproteins and glycolipids through transfer of sialic acids from CMP-sialic acid to the nonreducing ends of sialic acid acceptors [30]. Our previously showed that the sialyltransferase ST8SIA4, encoded N-acetylgalactosaminide a-2, 8-sialyltransferase IV (ST8SIA4), was critical for CML multidrug resistance [19, 20]. In addition, miRNAs that target glycosylation enzymes have been identified. Vaiana et al demonstrated that MGAT4A, an N-acetylglucosamintltransferase that installed the β-1, 4 branch of N-glycans, was directly regulated by miR-424 in multiple mammary epithelial cell lines [31]. MiR-200a might target ST3GAL3 and ST3GAL4 sialyltransferases, which potentially involved in antithrombin sialylation, were 85% lower in neonates in comparison with adults [32]. MiR-199a targeted ST6GAL1 and reduced both the sialylation and the protein level of Necl-2 [33]. Herein we demonstrated that miR-181c targets the 3′ UTR of the sialylation related gene ST8SIA4, suggesting the enzyme may play a role in cancer chemoresistance. Thus, further exploration of the interaction between miR-181c and ST8SIA4 in alternate MDR system of CML was warranted. We showed that downregulation of miR-181 contributes to high levels of ST8SIA4. While, forced expression of miR-181c resulted in a significant decrease of ST8SIA4. We also observed a significant inverse correlation between miR-181c and *ST8SIA4* levels in CML patients. Furthermore, silencing of the *ST8SIA4* gene reversed antagomiR-181c-mediated sensitivity of the K562 cells to ADR, VCR and Taxol. Overexpression of *ST8SIA4* in K562/ADR cells increased chemoresistance after miR-181c introduction. Taken together, our findings suggest that miR-181c mediates CML chemoresistance at least in part by functionally targeting *ST8SIA4*.

Numerous studies have demonstrated that activation of the PI3K/Akt signaling pathway was essential to the development and progression of most cancer types and associated with nearly all aspects of the malignant phenotype of cancer, such as uncontrolled proliferation, resistance to cell death, invasiveness, angiogenesis and metastasis [34, 35]. PI3K/AKT inhibition correlated down-regulation of NF-kappaB activity and inhibition P-gp function in murine lymphoma cell lines [36]. Inhibition of the PI3K/mTOR pathway was a promising therapeutic approach in patients with acute lymphoblastic leukemia [37]. Recent evidences have indicated that miRNAs were involved in the regulation of PI3K/AKT signaling pathways. For example, overexpression of miR-22 in CLL B cells switched on PI3K/AKT, leading to downregulation of p27 (-Kip1) and overexpression of Survivin and Ki-67 proteins [38]. Ectopic expression of miR-206 mimics inhibited cisplatin resistance, decreased the migration and invasion in cisplatin-resistant lung adenocarcinoma cells, partly due to inactivation of MET/PI3K/AKT/mTOR signaling pathway [39]. Our recent study showed that downregulation of *ST8SIA4* expression suppressed the activity of PI3K/AKT pathway. In the present study, we found miR-181c-*ST8SIA4* axis regualted PI3K/AKT in CML cell lines. MiR-181c inhibitor enhanced phosphorylation of PI3K/AKT in K562 cells. In contrast, miR-181c suppressed the phosphorylation of PI3K/AKT in K562/ADR cells. Further investigation detected Akt shRNA inhibited the phosphorylation of PI3K/AKT in K562-antagomiR-181c cells, subsequently leading to a decrease in chemoresistance. Other studies also reported that ectopic expression of miR-181a leads to AKT phosphorylation, enhancing cell proliferation and inducing cell resistance to chemotherapy in T-cell leukemia/lymphoma [40]. These findings may indicate that miR-181 functions as both an oncomir and tumor-suppressive miRNA, depending on the tumor type and the subtypes of miR-181. These results provides a possible mechanism linking miR-181c, PI3K/AKT pathway, drug resistance, by which altered expression of *ST8SIA4* leads to drug resistance in CML cell lines. In addition, we also assessed the anti-tumor effect of miR-181c in an adriamycin-resistant *in vivo* mice model. We found that overexpression of miR-181c enhanced the K562/ADR cell sensitivity to adriamycin and inhibited the PI3K/AKT pathway and *ST8SIA4* expression *in vivo*. Therefore, these results further demonstrated *in vivo* that miR-181c inhibiting *ST8SIA4* and its downstream PI3K/AKT pathways is one potential mechanism to overcome drug resistance in CML.

In conclusion, our study demonstrated that miR-181c was downregulated in adriamycin-resistant chronic myelocytic leukemia cell lines and CML/MDR patients. In addition, we showed that miR-181c regulated chemoresistance both *in vitro* and *in vivo* by targeting ST8SIA4 and mediating its downstream PI3K/AKT. Therefore, activation of miR-181c or inactivation of its target gene pathway may be a potential strategy to reverse drug resistance in human CML.

## MATERIALS AND METHODS

### Cell culture

Human CML in blast crisis cell lines, K562, KCL22, KU812 were both cultured in RPMI 1640 medium (Gibco, Grand Island, NY) supplemented with 10% fetal bovine serum (Gibco, Grand Island, NY) and 1% penicillin-streptomycin (Gibco, Grand Island, NY) at 37°C in humidified atmosphere containing 5% CO2. The resistant cell lines, K562/ADR, KCL22/ADR, and KU812/ADR were incubated in the presence of adriamycin (Sigma, St Louis, MO, 1 μg/ml) until at least 3 days before starting the experiments.

### Primary patient samples

Thirty-eight previously untreated CML patients were included in this study. All patients were diagnosed with CML from 2013 to 2015 at First Affiliated Hospital of Dalian Medical University (Dalian, China) according to the current World Health Organization criteria. The diagnosis of CML was based on cytomorphology, cytochemistry, multiparameter flow cytometry, immunology, molecular genetics and cytogenetics. All the participants were provided written informed consent and the study were approved by and the institutional ethics committees. Peripheral blood mononuclear cells (PBMC) were isolated using Ficoll-Hypaque and were further cultured in plastic dishes to remove adherent cells at 37°C for 24 h. The PBMC were divided into two groups, CML without MDR and CML/MDR. The frequency of P-gp positivity was 57.9% (22 of 38) in the CML patients. The clinical data of enrolled 38 patients was given in Supplementary Table S1.

### miRNA expression arrays

miRNA arrays were performed for K562 group and K562/ADR group (n=3/group) by Exiqon (KangChen, China) using the miRCURY™Hy3™/Hy5™ power labelling kit and the miRCURY™ LNA Array (v.10.0; 757 human miRs). The expression values are log2 (Hy3/Hy5) ratios. Unsupervised hierarchical clustering of miRNAs was performed.

### RNA extraction and real-time PCR

Total RNAs for array analysis were extracted using Trizol (Invitrogen) and RNAeasy mini kit (Qiagen) or RNA was precipitated with isopropanol and glycogen (Invitrogen) according to the manufacturer's instructions. cDNA were synthesised from RNA using a TaqMan miRNA reverse transcription kit (Applied Biosystems, Foster City, CA, USA), and quantified the expression levels of miR-181c using a miRNA-specific TaqMan MiRNA Assay Kit (Applied Biosystems). The expression of miRNA was defined based on the Ct, and relative expression levels were calculated as 2^−[(Ct of miR-181c) − (Ct of U6)]^ after normalization with reference to expression of U6 small nuclear RNA.

### Western blotting

Cells were harvested, lysed and sonicated in RIPA buffer. A total of 10-50 μg of protein was electrophoresed in SDS-PAGE gels and transferred to polyvinylidene difluoride membranes (Millipore, Bedford, MA, USA). The membranes were then incubated with the following antibodies: anti-ST8SIA4, anti-PI3K p110α, anti-phospho-Akt 308, anti-phospho-Akt 473, and anti-Akt and anti-NF-κB antibodies (Abgent, Cambridge, UK). The immunoblots were visualized using an enhanced chemiluminescence detection system (Amersham Biosciences, Buckinghamshire, UK).

### Cell viability assay

The cell viability was monitored using the Cell Counting Kit-8 (CCK8) (Dojindo Molecular Technologies, Kumamoto, Japan) according to the manufacturer's protocol. Briefly, cells (1×10^4^) were plated in 96-well plate and allowed to grow for 48 h before the addition of CCK8. The spectrometric absorbance was measured at 490nm by microplate reader (Model 680; Bio-Rad, Hercules, CA, USA). All of the experiments were repeated at least three times.

### Apoptosis assay

Cell apoptosis was evaluated using an Annexin-V-FITC apoptosis detection kit (BD, Franklin Lakes, NJ, USA). Briefly, after treatment with chemotherapeutic agents (K562/ADR, ADR/VCR/Taxol 40 μg/ml; K562, ADR/VCR/Taxol 1μg/ml), for 48 h, 2×10^5^ cells were harvested, resuspended in 100 μl flow cytometry binding buffer, and stained with 5 μl Annexin V/FITC followed by 1 μl PI. Cells were then incubated in the dark for 15 min at room temperature, and 400 μl binding buffer was added. The cells were immediately measured by FACSCalibur (Becton-Dickinson, CA, USA).

### Plasmids, oligonucleotides, shRNA and transfection

The region of the human *ST8SIA4* 3′-UTR, generated by PCR amplification from DNA, was cloned into vector pmirGLO (Promega). MiR-181c mimic, antagomiR-181c from RiboBio (Guangzhou, China). For depletion of *ST8SIA4*, shRNAs were synthesized and purified. Overexpressing of *ST8SIA4* was generated by transduction using a pEGFP-N2 vector (Invitrogen, Carlsbad, CA). Transfection was performed using the Lipofectamine 2000 reagent (Invitrogen), according to the manufacturer's instruction.

### Reporter gene assay

The pmirGLO-*ST8SIA4* 3′-untranslated region (UTR) luciferase-reporter (containg Firefly luciferase reporter gene and humanized Renilla luciferase) (Promega, Madison, WI, USA) containing many copies of the miR-181c binding motif was co-transfected with miR-181c mimics or mimic control into HEK 293T cells using Lipofectamine 2000 (Invitrogen). Luciferase and Renilla signals were measured 48 h after transfection using a Dual-Luciferase® Reporter Assay Kit (Promega) according to the manufacturer's protocol.

### Xenografted tumor model

Nude mice (4 weeks old) were purchased from the Animal Facility of Dalian Medical University Animal Facility of Dalian Medical University and housed in barrier facilities on a 12-h light/dark cycle. The mice were randomly assigned to groups (n = 6/group). The mice in groups were inoculated subcutaneously with K562/ADR cells (1×10^7^) in the right flank, and one week later, injected intratumorally with miR-181c mimic or mimic control three times per week for 3 weeks, combining with intraperitoneal injection of doxorubicin (7mg/kg) weekly. Tumors were examined every 7 days. Tumor volume was calculated using the equation (length × width^2^)/2.

### Immunohistochemistry

At the end of observation animals were sacrificed and tumors were retrieved for further analysis. Tumors were immediately immersed in 4% buffered formaldehyde, washed, dehydrated, and finally embedded in paraffin. Tumor slices 5 μm thick were deparaffinized. After washing steps, peroxidase blocking was carried out for 10 min to quench the endogenous peroxidase. Tumors were again washed and then incubated with the primary antibodies at 4°C overnight. The secondary streptavidin-horseradish peroxidase-conjugated antibody staining (Santa Cruz Biotech, Santa Cruz, CA) was performed at room temperature for 60 min. Subsequently, slides were counterstained with hematoxylin.

### Statistical analysis

The statistical analyses were performed using SPSS software. The data are presented as the mean± SD. Student's t-test (two-tailed) was employed to analyze the significance of differences between two groups of data in all pertinent experiments. The one-way analysis of variance (ANOVA) was used to determine whether there are any significant differences between the means of more than two groups. The association between miR-181c and *ST8SIA4* expression was evaluated using Spearman's correlation analysis. Statistical significance was defined as p< 0.05.

## SUPPLEMENTARY TABLE


