# Supplementary material for: Upregulation of miR-181c inhibits chemoresistance by targeting ST8SIA4 in chronic myelocytic leukemia
Source: Oncotarget. 2016 Aug 4;7(37):60074–86. doi: 10.18632/oncotarget.11054 (PMC5312369; doi:10.18632/oncotarget.11054)
Supplement: Supplementary file 1 [file oncotarget-07-60074-s001.pdf]

Upregulation of miR-181c inhibits chemoresistance by targeting *ST8SIA4* in chronic myelocytic leukemia

SUPPLEMENTARY TABLE

Supplementary Table S1: Clinicopathologic characteristics of the leukemia patients

Patients demographics	Subcategory	CML (n=38)
Gender	Male	23
	Female	15
Age (years)	Median	53
	Range	18-76
Splenic enlargement	_	33
Hemoglobin <100.0 g/L	_	12
WBC count (10^9/L)	20-100	22
	>100	16
Platelet count (10^9/L)	<300	17
	>300	21
P-gp (+)	_	22

Abbreviations: CML, chronic myelocytic leukemia; P-gp, P-glycoprotein; WBC, white blood cell.
